# Induction of Larval Metamorphosis of the Coral *Acropora millepora* by Tetrabromopyrrole Isolated from a *Pseudoalteromonas* Bacterium

**DOI:** 10.1371/journal.pone.0019082

**Published:** 2011-04-29

**Authors:** Jan Tebben, Dianne M. Tapiolas, Cherie A. Motti, David Abrego, Andrew P. Negri, Linda L. Blackall, Peter D. Steinberg, Tilmann Harder

**Affiliations:** 1 School of Biological, Earth and Environmental Sciences and Centre for Marine Bio-Innovation, University of New South Wales, Sydney, Australia; 2 Australian Institute of Marine Science, Townsville, Australia; 3 Sydney Institute of Marine Science, Mosman, Australia; King Abdullah University of Science and Technology, Saudi Arabia

## Abstract

The induction of larval attachment and metamorphosis of benthic marine invertebrates is widely considered to rely on habitat specific cues. While microbial biofilms on marine hard substrates have received considerable attention as specific signals for a wide and phylogenetically diverse array of marine invertebrates, the presumed chemical settlement signals produced by the bacteria have to date not been characterized. Here we isolated and fully characterized the first chemical signal from bacteria that induced larval metamorphosis of acroporid coral larvae (*Acropora millepora*). The metamorphic cue was identified as tetrabromopyrrole (TBP) in four bacterial *Pseudoalteromonas* strains among a culture library of 225 isolates obtained from the crustose coralline algae *Neogoniolithon fosliei* and *Hydrolithon onkodes*. Coral planulae transformed into fully developed polyps within 6 h, but only a small proportion of these polyps attached to the substratum. The biofilm cell density of the four bacterial strains had no influence on the ratio of attached vs. non-attached polyps. Larval bioassays with ethanolic extracts of the bacterial isolates, as well as synthetic TBP resulted in consistent responses of coral planulae to various doses of TBP. The lowest bacterial density of one of the *Pseudoalteromonas* strains which induced metamorphosis was 7,000 cells mm^−2^ in laboratory assays, which is on the order of 0.1 –1% of the total numbers of bacteria typically found on such surfaces. These results, in which an actual cue from bacteria has been characterized for the first time, contribute significantly towards understanding the complex process of acroporid coral larval settlement mediated through epibiotic microbial biofilms on crustose coralline algae.

## Introduction

Chemical signals play critical roles at every organisational level in marine systems and constitute much of the language of life in the sea [1]. For many sessile marine invertebrates (e.g. sponges, corals, mussels) chemical signals play a major role in determining the choice of habitats in which juveniles establish themselves. Many benthic marine invertebrates have a motile, planktonic larval phase, and the transition between larvae and the less mobile or immobile benthic stage is marked by a metamorphic event. Because larval attachment and metamorphosis into sessile juveniles is generally irreversible [2], the signalling of suitable locations for settlement by invertebrate larvae is critical to the population and community dynamics of these organisms.

Despite the importance of chemical cues for larval settlement, their actual identities are poorly described, and only very few elicitors have been fully chemically characterized and assigned to a defined natural source [3], [4], [5], [6], [7]. Indeed, to our knowledge only three studies [3], [5], [8] have both characterized chemical cues and supported their ecological role by having verified their production, presence and/or release at ecologically realistic concentrations. This significantly constrains our ability to understand the role such signals play in benthic community dynamics. Moreover, because the induction of larval attachment and metamorphosis of benthic marine invertebrates is widely considered to rely on receptor-mediated processes [9], our lack of knowledge of cue identity also means that our understanding of the mechanistic processes involved in site recognition and induction of metamorphic cascades is poor.

Marine hard substrata are coated with ubiquitous microbial biofilms, and these have received considerable attention as habitat specific settlement signals for a wide and phylogenetically diverse array of marine invertebrates [9], [10], [11], [12], [13], [14]. Detailed investigations with a marine polychaete, *Hydroides elegans*, a model organism for studying larval responses to biofilms, have shown that stimulation of larval attachment and metamorphosis by monospecies bacterial biofilms is both species-specific and cell density dependent [15]. A fatty acid and a simple hydrocarbon in extracts of natural biofilms induced larval settlement in *H. elegans* [16]; however the source of these signals, out of the many possible organisms in the biofilm (complex agglomerates of bacteria, protozoa, and microalgae [17]), was not determined. Thus, despite the long recognized role of marine bacterial biofilms as potent inducers of larval attachment and metamorphosis [18], no signaling molecule which induces metamorphosis has yet been characterized from a marine bacterium, severely limiting our capacity to understand the role of bacteria in mediating population and community dynamics of marine invertebrates.

The larvae of tropical hard corals are classic examples of organisms that selectively settle in response to habitat specific cues such as crustose coralline algae (CCA) [14] and epiphytic bacterial biofilms associated with these algae [19], [20], [21]. However, settlement responses of coral larvae to biofilm bacteria vary greatly among bacterial isolates. For example, Negri et al. [20] found that only one of 20 bacterial isolates from the CCA *Hydrolithon onkodes* (*Pseudoalteromonas* strain A3) induced larval metamorphosis of the abundant reef-building corals *Acropora millepora* and *A. willisae*.

Our aims in this study were to investigate whether bacterial induction of larval metamorphosis of coral larvae was chemically mediated and to isolate and identify putative chemical metamorphic inducers from *Pseudoalteromonas* strain A3 and other biofilm bacteria in coral reef habitats. This was done by screening 200 bacterial isolates from a highly inductive and widely abundant CCA in the Great Barrier Reef (GBR), *Neogoniolithon fosliei*, in coral larval settlement assays, followed by bioassay guided fractionation of inductive strains in order to characterise any chemical cues of bacterial origin.

The coral *A. millepora* was used in this study because it is an important reef building coral in the GBR and its genome has just been mapped [22]. Hence, the knowledge of chemical signals responsible for larval metamorphosis in this coral represents a crucial piece of information to elucidate the underlying mechanisms of coral larval settlement on a molecular, cellular, and genomic level in the future.

## Results

### Screening and phylogenetic identification of single strain bacterial films

Of 200 distinguishable morphotypes isolated from the CCA *N. fosliei*, three yellow pigmented strains J010 (AN JF309049), J021 (AN JF314511) and J104 (JF314512) induced metamorphosis in 100% of exposed larvae. The other 197 bacterial isolates had no effect on larval metamorphosis, relative to controls, when tested as single strain biofilms, regardless of the presence or absence of sterile chips of *Porites* sp. In the presence of J010, J021 and J104, larvae flattened into discs and displayed obvious septal mesenteries radiating from the central mouth region, indicating a significant developmental event similar to that observed by Negri et al. [20]. However, most of these newly formed polyps remained floating at the water surface, with very few (max. 10% over all replicates) permanently attached to the dish. An identical response was observed in the presence of *Pseudoalteromonas* strain A3. In contrast, settlement (metamorphosis and attachment) was observed in treatments containing live CCA chips of *N. fosliei* and *H. onkodes.* The potent effect of isolates J010 and J021 on metamorphosis was reproducibly observed after storage and inoculation from the frozen glycerol stock.

**Figure 1:**
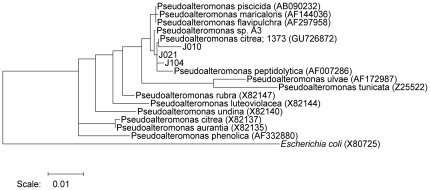
Phylogenetic tree of bacteria affiliated to the genus *Pseudoalteromonas* based on 16S rRNA gene sequences (5′-prime region, poisitions 10 to 509 *E. coli* equivalent). Nucleotide distances are based on the maximum likelihood algorithm and the tree clustered using the Neighborjoining procedure.

Based on their 16S rDNA sequences, isolates J010, J021, and J104 were phylogenetically highly similar to each other (>99.5%) and affiliated most closely with *Pseudoalteromonas citrea* 1373 (GU726872) and *Pseudoalteromonas peptidolytica* (AF007286) (Fig. 1). They shared more than 99.3% similarity with *Pseudoalteromonas* strain A3 [20]. Due to their high similarity only J010 was used for the bioassay-guided isolation of the metamorphic cue.

### Effect of bacterial biofilm density on larval settlement

**Figure 2:**
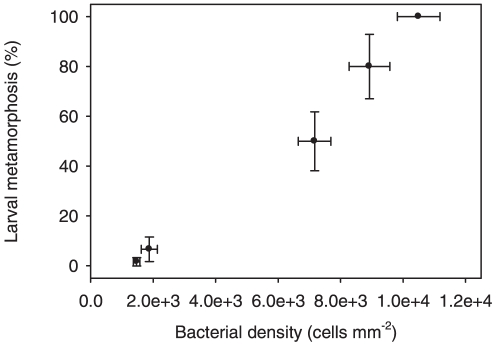
Percentage of larvae undergoing metamorphosis in response to biofilms of *Pseudoalteromonas* strain J010 at a range of bacterial densities. Each data point represents the mean percentage of metamorphosis (±SE) of 6 replicates containing 10 larvae each, and the mean bacterial density (±SE) of 6 replicates with 10 cell counts in each replicate.

There was a fine scaled and statistically significant effect of the biofilm density of strain J010 on the induction of metamorphosis of coral larvae (F = 34, p = 0.001, permutational ANOVA; Fig. 2). The lowest cell density to induce significant levels of metamorphosis was 7200±520 cells mm^−2^ and at this density 50±12% of larvae had undergone metamorphosis (F = 4.2, p = 0.002, pair-wise comparison to the negative control). Bacterial densities of 10,500±680 cells mm^−2^ induced metamorphosis in 100% of larvae.

### Bioassay-guided isolation of the metamorphic cue

**Figure 3:**
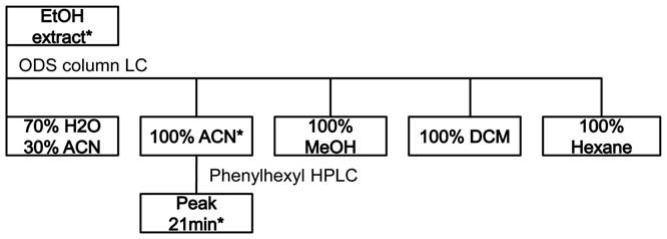
Isolation of the bacterial metabolite of *Pseudoalteromonas* strains J010 that induced metamorphosis of coral larvae. The asterisk marks 100% larval metamorphosis in bioassays after 6 h.

**Figure 4:**
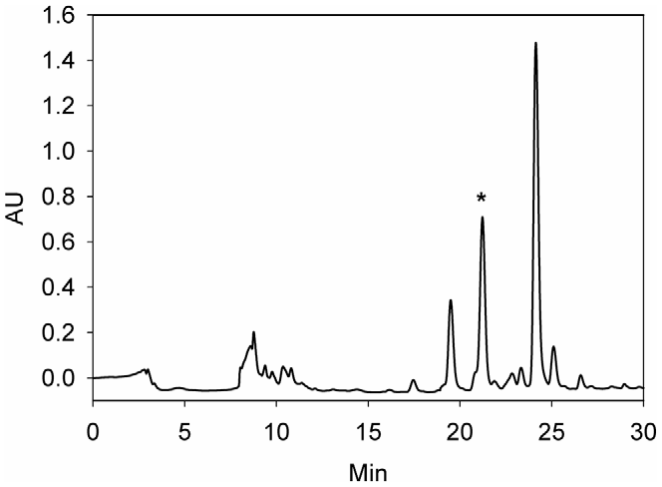
HPLC chromatogram of the inductive C18-chromatographic fraction of *Pseudoalteromonas* strain J010. The asterisk marks the peak fraction that induced 100% metamorphosis of coral larvae.

A cue which induced metamorphosis was successfully isolated from the ethanol extract of J010 by bio-assay guided fractionation. The inductive fraction that triggered 100% of larval metamorphosis eluted with 100% acetonitrile from a C_18_-flash column (Fig. 3). This eluate was concentrated and purified by high performance liquid chromatography (HPLC) to give a single active fraction at RT = 21 min (Fig. 4).

### Structure elucidation and biological activity of synthetic tetrabromopyrrole

Analysis of the low resolution mass spectrum of the purified metamorphosis inducing cue identified peaks at *m/z* 377.9, 379.9, 381.7, 383.6, 385.6 (1∶4∶6∶4∶1) indicative of a tetrabrominated compound. This compound was confirmed as tetrabromopyrrole (TBP) by comparison of the ^1^H and^ 13^C NMR and FTMS spectral data (observed *m/z* 377.6771, calculated for C_4_NBr_4_^−^, *m/z* 377.6770) with literature values [23]. TBP is unstable and extremely light sensitive [24] and contact to air and light was avoided where possible during later isolations from bacterial biofilms.

TBP was chemically synthesized and HPLC of the crude reaction product confirmed the presence of a peak at RT = 21 min, corresponding to that of natural TBP isolated from the ethanol extract of J010. The synthetic TBP was isolated from other reaction products and NMR and MS spectral data were confirmed to be identical to natural TBP.

**Figure 5:**
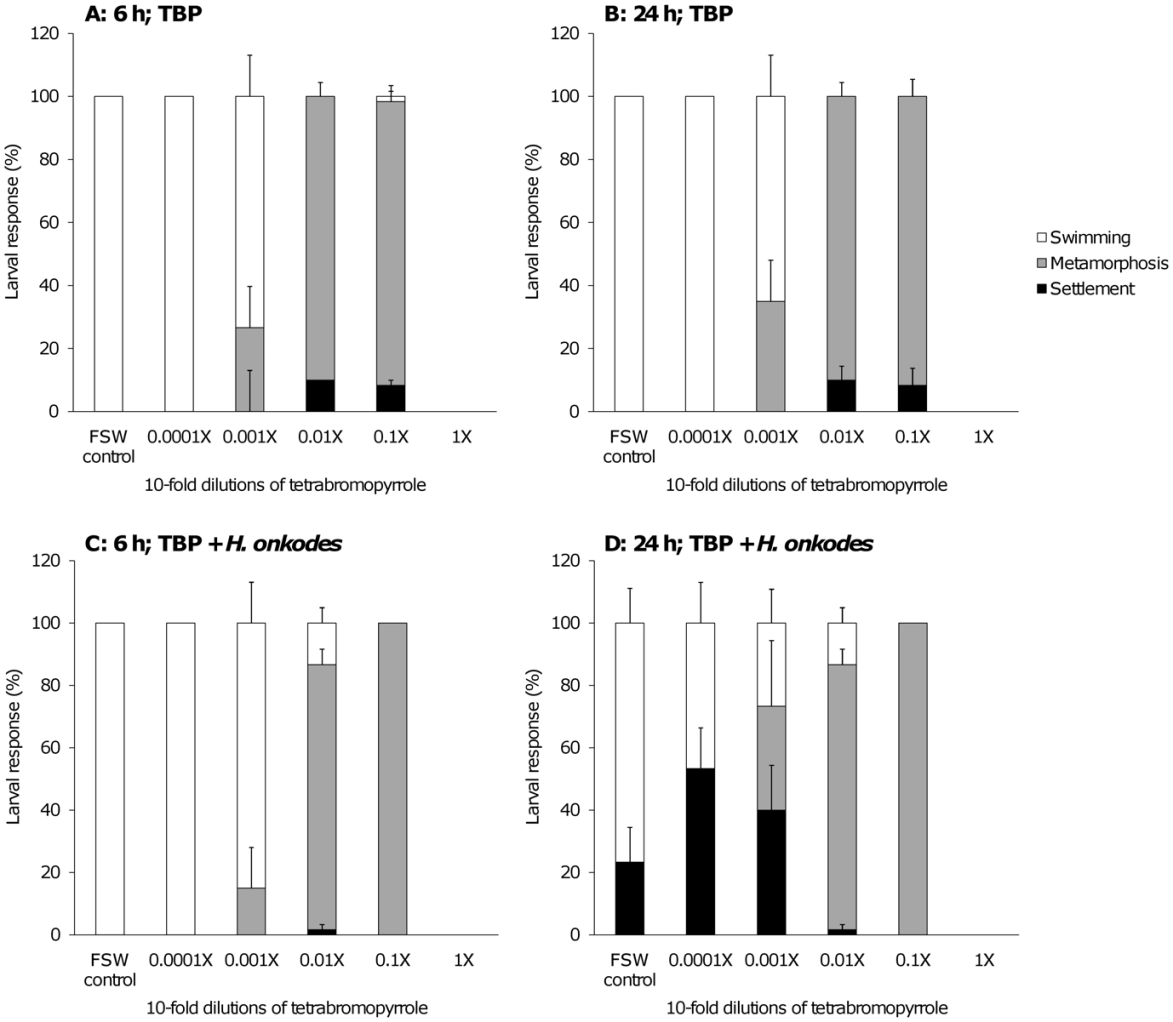
Different types of larval response (settlement, metamorphosis, swimming) to a 10-fold dilution series of synthetic tetrabromopyrrole in absence (A, B) and presence (C, D) of chips of *Hydrolithon onkodes* after 6 h (A, C) and 24 h (B, D). The control contained FSW only. The 1x concentration was lethal to the larvae. Each value (error bars) represents the mean (±SE) of 6 replicates with 10 larvae in each replicate.

Synthetic TBP induced metamorphosis of *A. millepora* larvae after 6 h (F = 48, p = 0.001, permutational ANOVA) (Fig. 5A). The 1∶10 and 1∶100 dilution of synthetic TBP resulted in the same levels of metamorphosis (∼90%) after 6 h and were different from the control (for 1∶10; F = 17, p = 0.001; for 1∶100; F = 20, p = 0.001, pair-wise comparison to the negative control). The stock solution was lethal to 100% of coral larvae after 6 h. The proportion of larvae metamorphosed at each TBP dilution was not affected by the presence of live CCA (*H. onkodes*) after 6 h (F = 0.7, p = 0.545, 2-way-permutational ANOVA; Fig. 5A, C). There was no further metamorphosis observed following a further 18 h exposure to TBP at each of the dilutions in the absence of live CCA (F = 0, p = 1, 2-way-permutational ANOVA; Fig. 5A, B) and in the presence of live CCA (p = 0.991, 2-way-permutational ANOVA; Fig. 5C, D).

The presence of live CCA chips induced a significant settlement response (metamorphosis+attachment) after 24 h in the TBP dilution of 1∶1000 (40±14%) and 1∶10,000 (53±13%) as well as in the control (23±11%) (Fig. 5D) compared with the same dilution series after 6 h (F = 17, p = 0.001, permutational ANOVA; Fig. 5C, D). However, little attachment (<10% was observed in the absence of live CCA after 6 and 24 h (Fig. 5A, B). These results show that larval metamorphosis triggered by TBP generally occurred within 6 h of exposure (Fig. 5A, B), whereas settlement of larvae in response to the CCA *H. onkodes* took as long as 24 h (Fig. 3 6D). Only those larvae that had not been triggered to metamorphose after 6 h (by TBP) were subsequently induced to settle in response to live CCA.

### Effect of growth conditions on induction of larval metamorphosis by isolate J010

The inductive effect of A3 and J010 was dependent on the type of growth medium (liquid vs. solid) and the presence of potassium bromide (KBr). The nutrient composition of the media did not affect the inductive effect of the ethanolic extracts of J010 as long as this strain was grown on solid media prepared with artificial seawater containing KBr. In contrast, no larval metamorphosis was observed when cells of J010 or A3 were grown in liquid suspension cultures or in the absence of KBr. The induction of larval metamorphosis by extracts of J010 coincided with the detection of the characteristic HPLC peak for TBP in ethanol extracts of J010 grown on different solid media in the presence of KBr. No such peak was observed from extracts of growth media lacking KBr. Ethanol extracts of J010 grown on media lacking KBr had significantly less yellow pigmentation, did not induce larval metamorphosis and did not contain detectable concentrations of TBP.

### Qualitative analysis of TBP in closely related bacteria to J010

The characteristic mass and spectral isotope pattern of TBP (1∶4∶6∶4∶1) was only observed in extracts of A3, J010 and J021, and not in extracts from any of the phylogenetically closely related strains *P. aurantia, P. citrea, P. luteociolacea, P. piscicida, P. tunicata, P. ulvae,* or *P. undina*.

## Discussion

It is well established that bacterial biofilms are widespread inducers of marine invertebrate larval attachment and metamorphosis [9], [18]. However, the isolation and characterization of the putative inductive chemical signals from bacteria has proved elusive. Here we report the isolation and elucidation of the first chemical signal from a known bacterium that induces transition of planulae into fully developed polyps in the acroporid coral *Acropora millepora*. This metamorphic cue was identified as tetrabromopyrrole (TBP) in three *Pseudoalteromonads* isolated from the crustose coralline alga (CCA) *Neogoniolithon fosliei*. This metamorphic cue was also the bacterial metabolite from the closely related *Pseudoalteromonas* strain A3 that induced metamorphosis in *A. millepora* as reported by Negri et al. [20].

The combined findings of this study and Negri et al. [20] suggest that only a very few (4 out of 220) metabolically unique bacteria on the CCA *N. fosliei and Hydrolithon onkodes* induce larval metamorphosis in *A. millepora*. Their uniqueness was further highlighted by the finding that TBP was not detected in other closely related *Pseudoalteromonas* strains in this study, although congeners such as *P. luteoviolacea* are known to produce other brominated pyrrole derivatives [25]. Prior to this study, TBP had only been described in a *Chromobacter* sp. [23]. TBP was only produced when the inductive bacteria were grown on surfaces as a biofilm and not when the bacteria were grown in suspension. Such differences in gene transcription are well known for bacteria grown in suspension vs. when grown as biofilms [26].

Despite the rapid metamorphic response of coral larvae to *Pseudoalteromonads* A3, J010, J104 and J021, 90% of the newly formed polyps did not firmly attach, and this was independent of the cell density in the bacterial biofilms. Larval metamorphosis without attachment has been observed in a phylogenetic diverse range of corals [27], [28] and was also described by Negri et al. [20], who observed that the majority of planulae, despite flattening into discs and developing septal mesenteries radiating from the central mouth region when confronted with a biofilm of *Pseudoalteromonas* strain A3, did not attach. Negri et al. [20] noticed that up to 50% of the newly formed polyps attached to the dish surface in the presence of the calcareous matrix of the coral *Porites* sp. and hypothesized that the calcareous matrix played a role in the synthesis of additional inducers of larval settlement of strain *Pseudoalteromonas* strain A3. However, we were not able to achieve comparable results with bacterial biofilms from *Pseudoalteromonas* strains A3 and J010 grown on sterile chips of *Porites* sp. skeleton (data not shown). The complete onset of larval attachment and metamorphosis was only observed in the presence of the CCA *N. fosliei* (data not shown) and *H. onkodes*.

The strong metamorphic effect of TBP on coral larvae was further highlighted by studying induction in the presence and absence of live CCA at different time intervals (6 and 24 h, Fig. 5). The metamorphic response of larvae to TBP was much faster than the settlement response to CCA only, which typically took 12 to 24 h to induce attachment and metamorphosis. In experiments with both TBP and CCA, only those larvae that had not been triggered to metamorphose after 6 h, such as in the 1∶1,000 dilution of synthetic TBP (Fig. 5C), attached and metamorphosed in response to CCA (Fig. 5D). At higher concentrations of TBP (>1∶100 dilution) larval attachment was seemingly short-circuited by the fast metamorphic response to TBP (Fig. 5C, D).

These outcomes suggest that complete settlement–larval attachment and metamorphosis–was unlikely to result from a synergistic action of TBP and another, as yet unknown chemical cue. Live CCA are consistently very strong inducers of complete larval settlement in acroporid corals (our study), and [27], [29], [30], both in the field or in less ecologically realistic Petri dish assays in the laboratory. However, our laboratory assays repeatedly showed that exposure of coral larvae to TBP–by itself and when added in the presence of live and inductive chips of CCA–resulted in unattached polyps. While there may be other as yet unidentified environmental co-factors in the field not present in our experiments which might reverse this effect (and thus result in complete settlement), our results suggest that TBP may not act as an inducer of complete settlement in the field, but is more likely to act as an antagonistic cue to full settlement.

The hypothesis that TBP short-circuits the natural settlement response of coral larvae to CCA is consistent with a conceptual model of the induction of larval metamorphosis in hydroids [31]. One of the characteristics of this model is that the natural bacterial inducer of metamorphosis can be side-stepped by the external exposure of larvae to a group of neuropeptides [32], which have been identified as the internal signalling molecules in the larval body [31]. One member of the neuropeptide family, Hym-248, also induced larval metamorphosis of *Acropora* spp. within 3–6 h, but the majority of larvae did not attach but rather floated at the water-air interface [27]. In terms of timing and behaviour, this response was very similar to the one observed to TBP in our study (Fig. 5A). This comparatively fast response was in contrast to the general 12–24 h required for attachment and metamorphosis in response to live CCA [20], [30] and suggested that the trigger of larval attachment is either separate from metamorphosis per se, or is bypassed [33].

This concept suggests that TBP may effectively act as an antifouling compound against coral larvae. Interestingly, TBP as a brominated metabolite fits into a category of other well known and potent defense compounds in the marine environment [34], [35]. In one case, compounds belonging to the same compound class as TBP (brominated pyrroles) inhibit settlement of barnacle cyprids [36].

One important caveat to this model of interference of signaling cascades and hence attachment (or indeed to any inference about the ecological effects of TBP) is that although we have repeatedly isolated TBP producing bacteria from two different species of CCA, we currently do not know the densities of the inductive strains on CCA in the field nor the concentrations of TBP *in situ.* Isolate J010 induced significant levels of metamorphosis at cell densities as low as 7,000 cells mm^−2^ (Fig. 2), and comparable densities for at least individual genera of bacteria, including *Pseudoalteromonas*, do occur on seaweeds in the field [37]. Still, the assessment of the ecological role of TBP crucially depends on its natural concentration. Until we have developed targeted and selective molecular and chemical tools to quantify the TBP-producing bacteria as well as the surface concentration of TBP on CCA in the field (e.g. by tools such as fluorescence in-situ hybridization (FISH) or desorption electrospray ionization mass spectroscopy (DESI- MS)), the ecological role of TBP will remain uncertain.

In summary, TBP is the first characterized bacterial inducer of larval metamorphosis in a marine invertebrate. The knowledge of chemical signals for larval attachment and metamorphosis is not only important for the field of marine chemical ecology, but has widespread implications for other fields such as aquaculture (to facilitate settlement of commercially valuable species), biofouling (inhibit settlement of fouling organisms) and reef restoration (rehabilitation of coral reefs *via* targeted larval settlement).

## Methods

### Spawning and culturing of coral larvae

Colonies of the scleractinian coral *Acropora millepora* were collected three to five days prior to predicted spawning events from two sites on the Great Barrier Reef (GBR) (November 2009 at Orpheus Island, 18°37′ S 146°28′ E and December 2009 at Trunk Reef, 18°22′ S, 146°47′ E) and one site at Ningaloo Reef (April 2010 at Coral Bay, 23°10′ S 113°45′ E). Three to five colonies were collected from each reef. Colonies from the GBR were transported to the Australian Institute of Marine Science (Townsville, QLD) for spawning and larval rearing. These colonies were maintained in outdoor tanks (1000 l) with flow-through seawater at ambient temperature (approximately 28°C). Colonies from Ningaloo Reef were transported to Coral Bay for spawning and larval rearing at the Coral Bay Research Station. These colonies were kept on temporary racks on the reef at Coral Bay. In all cases, colonies were isolated a few hours before the predicted spawning in 60–100 l tanks containing filtered seawater (FSW, 1 µm). Gametes were collected from the water surface after spawning and gently mixed in separate tanks with FSW for fertilization. After∼3 h, fertilized embryos were removed from the fertilization tanks and transferred to larval rearing tanks (300–500 l with flow-through FSW (0.2 µm GBR, 1 WA µm)), where they were maintained in low densities (∼1 larvae ml^−1^) until development of fully competent larvae. Full competence was identified when larvae elongated and became increasingly active, exhibiting demersal swimming and benthic searching behavior. Larvae from the GBR became fully competent 4–6 d after fertilization, whereas larvae from Ningaloo Reef took slightly longer (5–8 d).

### Larval bioassays

Larval bioassays were performed in sterile 6-well culture plates at 27–28°C. CCA were collected together with corals at both sites and maintained in flow-through seawater tables. Bioassays were performed with competent larvae 10 d (GBR) or 13 d (WA) post fertilization. Coral larvae (n = 10) were introduced to each well in 10 ml of FSW. Larval responses were recorded at 6 and after 24 h under the dissecting microscope and categorized as follows: a) swimming (swimming or crawling, elongated body shape), b) metamorphosis (flattened along the oral-aboral axis with clear radial subdivisions of mesenteries), c) settlement (metamorphosis plus attachment to the dish surface or CCA).

### Bacterial isolation from the CCA *Neogoniolithon fosliei*

The top layer (0.5 mm) of three living colonies of *N. fosliei* collected from the Davies reef (GBR) was scraped off with a sterile scalpel and suspended in sterile FSW. The suspension was vortexed for 10 min and serially diluted 1∶10 to 10^−6^. A 100 µl aliquot of each dilution was spread-plated in triplicate on 100% and 10% Marine Agar and incubated at 28°C for 72 h. Each distinguishable bacterial morphotype was re-streaked three times to achieve pure strains which were stored in 30% glycerol/70% Marine Broth at − 80°C.

### Development and screening of single-strain bacterial films for larval bioassays

Single colonies of each bacterial isolate (n = 200) were inoculated into 6-well culture plates containing Marine Broth and incubated for 12 h at 28°C. The broth was discarded and the resulting biofilms, clearly distinguishable as a turbid layer at the dish surface, were carefully rinsed with FSW (0.2 µm) in a reproducible manner. Each bacterial isolate was tested in duplicate both with and without sterile chips of *Porites* sp. (dead skeleton fragments). This screening experiment including all bacterial isolates was repeated twice. *Pseudoalteromonas* strain A3 was used as a positive control [20]. Larval responses were scored as described above. Treatments containing bacterial isolates that resulted in swimming larvae were scored as “non-inductive bacterial isolates”, whereas treatments containing mainly metamorphosed and settled larvae were scored as “inductive bacterial isolates”.

### Effect of bacterial density in larval bioassays

The effect of bacterial biofilm density on the magnitude of the larval response was investigated with the inductive strain J010. Briefly, ca. ten bacterial colonies were scraped from agar plates and suspended in 15 ml of FSW. This stock suspension was diluted 1∶1 with FSW (0.2 µm) in four consecutive steps resulting in five serial dilutions (1, 1∶2, 1∶4, 1∶8, 1∶16). To develop bacterial biofilms of different densities on glass cover slips, 500 µl aliquots of each dilution were added to microscope cover slips (n = 12) and incubated at room temperature for 4 h. Biofilm-coated cover slips were carefully rinsed in FSW and used directly in the larval settlement assay (n = 6) and scored after 12 h. The remaining 6 biofilm-coated cover slips obtained from each dilution were stored in FSW (0.2 µm) without larvae for the duration of the bioassay. Subsequently, they were fixed in 10% formaldehyde in FSW, washed with phosphate buffered saline (PBS) and stained with 100 ng ml^−1^ of 4*′*, 6*-*diamidino*-*2*-*phenylindole in glycerol: water (1∶1) for 12 min. Biofilm-coated cover slips were photographed ten times under a fluorescence-microscope. The bacterial cell density was recorded with ImageJ v 1.43 software [38].

### Phylogenetic affiliation of bacteria

The 16S rRNA genes of pure bacterial isolates were amplified using colony PCR. The top of a single colony was touched with a sterile toothpick and transferred to an Eppendorf tube with 50 µl of TE buffer (10 mM Tris, pH 8.0; 1 mM EDTA). Each sample was heated to 95°C for 3 min then held at 4°C and briefly spun in a microfuge to pellet the cell debris. A 0.5 µl aliquot of the supernatant was used as a template for 20 µl PCR reactions containing 12.5 µl Econotaq 2x mastermix, 10 pmol each of 27F (AGAGTTTGATCCTGGCTCAG) and 1492R (GGTTACCTTGTTACGACTT) primers and 10 µl of deionized water. The PCR protocol included initial denaturation at 94°C for 3 min, 25 cycles of heating (94°C for 30 s, 55°C for 45 s and 72°C for 2 min), followed by a final extension at 72°C for 5 min and storage at 4°C. PCR amplicons were checked for correct size on 1% sodium borate agarose gel (250 V for 10 min) and purified using silica spin columns (DNA concentrator 5, Zymo Research). The amplicon concentration was measured spectrophotometrically. Purified PCR product (10 ng) was sequenced bi-directionally using Big Dye v3.1 (Applied Biosystems) with modifications to manufacturers' instructions. Each reaction contained 0.5 µl Big Dye Terminator, 0.75 µl 5x buffer, 5 pmol of primer (27F or 1492R) and 10 µl of deionized water. Forward and reverse sequences were combined using CAP3 [39]. Sequence reads were automatically trimmed to remove poorly resolved base pairs as determined by quality scores. The reverse compliments of these sequences were then aligned to give a consensus sequence. Sequences were checked against the ribosomal database project (RDP) 12 and a species tree constructed using RPDs tree building function with closely related type strains included for comparison.

### Bioassay-guided isolation of the metamorphic cue

The metamorphic cue of the inductive bacterial strain J010 was isolated by bioassay-guided fractionation. Two hundred Marine Agar plates were inoculated with a stock culture of J010 and grown for 24 h at 28°C. Bacterial colonies were carefully scraped off and the pellet (∼20 g bacterial biomass) was extracted twice with ethanol. The combined extract was subjected to chromatography on a reversed phase C_18_-flash vacuum column (5×100 cm) and sequentially eluted with (water/acetonitrile 70∶30, acetonitrile, methanol, dichloromethane and hexane). The resulting five fractions were tested in the bioassay. The inductive fraction, eluted with 100% acetonitrile, was concentrated and further purified on a Shimadzu HPLC system consisting of a SCL-10Avp system controller equipped with a LC-10AT pump, a SPD-M10Avp photodiode array detector, a FRC-10A fraction collector, and a SIL-10A auto sampler run on Class-VP software. Separation was achieved by elution with an acetonitrile-water gradient (70% at 0 min, held for 3 min and then rising linearly to 100% at 23 min) at a flow rate of 6 ml min^−1^ from a Luna phenyl-hexyl column (Phenomenex, 250×21.2 mm, 5 µm). The chromatogram was monitored at λ 220 nm. Low resolution mass spectral data of the inductive fractions were also measured by direct infusion on a Bruker Daltonics Esquire 3000 ion-trap mass spectrometer (MS) with an Apollo electrospray ionization (ESI) source operating in negative mode.

### Structure elucidation

Structure elucidation of the isolated metamorphic cue was achieved using Nuclear Magnetic Resonance (NMR) spectroscopy and Fourier Transform Mass Spectrometry (FTMS). ^1^H and ^13^C NMR spectra were acquired employing a Bruker Avance 600 MHz NMR spectrometer with cryoprobe. NMR spectra were referenced to residual ^1^H and ^13^C resonances in the deuterated solvents. Both 1- and 2-dimensional NMR spectra were recorded using standard Bruker pulse sequences. High resolution mass spectra were measured with a Bruker BioApex 47e FTMS fitted with an Analytica of Branford ESI source; ions were detected in negative mode within a mass range *m*/*z* 200–1,000. Direct infusion of fractions and pure compounds was carried out using a Cole Palmer 74900 syringe pump at a flow rate of 120 *µ*l h^−1^. The instrument was calibrated with methanolic trifluoroacetic acid (0.1 mg ml^−1^).

### Chemical synthesis of tetrabromopyrrole and its use in larval bioassays

Tetrabromopyrrole (TBP), the characterized inducer, was synthesized according to Palmer [24] and purified from the reaction mixture using the same chromatographic conditions described above for the extract of bacterial strain J010. A TBP stock solution of unknown concentration was dried by rotary evaporation in the dark, immediately dissolved in dimethylsuloxide (DMSO), and stored at 4°C until use in bioassays. As TBP is unstable and extremely light sensitive [24], especially as a solid, the preparation of stock solutions of known concentrations was not possible. Instead, a serial dilution (1, 1∶10, 1∶100, 1∶1000 and 1∶10000) of the purified synthetic TBP was prepared in DMSO. These dilutions were tested for the induction of larval metamorphosis (10 larvae per well) with replication (n = 6) in FSW making sure that the final concentration of DMSO in the assay did not exceed 0.01% (v/v). This experiment was performed in the absence and presence of live *H. onkodes* chips.

### Effect of growth conditions on induction of larval metamorphosis by isolate J010

Aliquots of a bacterial stock culture of the inductive strains J010 and A3 were each inoculated on four different nutrient agars (1.5% agar) and in four different liquid nutrient media, all of which were duplicated in presence or absence of potassium bromide (0.053 g l^−1^ artificial seawater). This approach tested the effect of growth substrate and the requirement of potassium bromide for these bacteria to produce an inducer for larval metamorphosis. Media were prepared in 4 combinations containing a) glucose (0.5 g l^−1^), yeast (5 g l^−1^), peptone (3 g l^−1^); b.) glucose (0.5 g l^−1^), tryptone (3 g l^−1^); c.) glucose (0.5 g l^−1^), peptone (3 g l^−1^); d.) glucose (0.5 g l^−1^), yeast (5 g l^−1^). Subsequently, 1 mg of bacterial biomass/pellet obtained from each of the different nutrient agars/broth cultures was extracted in 1 ml of ethanol. These extracts (5 µl extract in 10 ml FSW) were tested in larval bioassays and also analysed by HPLC for the presence of TBP. The cell-free broth supernatants were processed through C_18_ SPE mini columns and analysed accordingly by HPLC for the presence of TBP.

### Qualitative analysis of tetrabromopyrrole in bacteria closely related to strain J010

A number of *Pseudoalteromonas* species closely related to inductive strain J010 (*Pseudoalteromonas* strain A3, *P. aurantia, P. citrea, P. luteoviolacea, P. piscicida, P. tunicata, P. ulvae,* and *P. undina)* were grown on Marine Agar and extracted as described above for strain J010. Extracts were processed using C_18_ SPE mini chromatography columns and the eluates analyzed for the presence of TBP by direct infusion on a LTQ Orbitrap nano-ESI mass spectrometer (Thermo) operating in negative high mass resolution mode.

### Statistical analysis and data treatment

Most data did not fulfil the conditions of normality and homoscedasticity and could not be improved by transformation. Data were analyzed by permutational analysis of variance in the PERMANOVA routine of PRIMER v6 [40]. PERMANOVA relies on comparing the observed value of a test statistic (F-ratio) against a recalculated test statistic generated from random permutation of the data. The stated advantage of the permutation approach is that the resulting test is “distribution free” and not constrained by many of the typical assumptions of parametric statistics. PERMANOVA with 999 permutations based on Euclidean distance followed by pairwise comparisons was used to statistically evaluate experimental treatments.
